# Impact of COVID-19 on the Italian Mental Health System: A Narrative Review

**DOI:** 10.1093/schizbullopen/sgac038

**Published:** 2022-10-18

**Authors:** Bernardo Carpiniello, Antonio Vita

**Affiliations:** Department of Medical Sciences and Public Health, University of Cagliari, Cagliari, Italy; Department of Clinical and Experimental Sciences, University of Brescia, Brescia, Italy

**Keywords:** COVID-19, pandemic, Italy, mental health system

## Abstract

Italy has been severely affected by the COVID-19 pandemic, consequently producing a heavy burden on the Italian National Health Service. From February 2020 until the end of the same year, the Italian Mental Health System (MHS), comprising an extensive network of community services, was subjected to a significant decrease in standards of care followed at the beginning of 2021 by a slow return to usual levels of activity. Data reported in the present article highlight how the Italian MHS – as was the case in the majority of countries—was largely unprepared for this emergency, suggesting an impelling need to develop appropriate supplementary national plans with the aim of preventing similar situations from developing in the future. The upheaval caused by the pandemic has highlighted the need to reinforce, both at a local and national level, the organization and standards of care of the Italian MHS in order to protect and support the mental health of patients with severe mental disorders, health workers, and the general population, thus preventing a potential “pandemic” of mental disorders.

## Introduction

Italy was the first Western country to be severely affected by the COVID-19 pandemic. At the start of the first wave, on April 17, 2020, the country reported 159 107 confirmed cases and 19 996 deaths, with an overall fatality rate of 12.6%.^1^ To date, numbers have remained impressive: on February 1, 2022, according to the World Health Organization (WHO),^2^ Italy continues to rank third amongst the European Regions, following the Russian Federation and United Kingdom, for number of deaths. The present article provides an overview based on recent literature of the impact of the COVID-19 Pandemic on the Italian Mental Health System.

## Organization of the Italian Mental Health System

The transition from a hospital-centric to a community mental health-focused care system was first implemented in Italy by a reform dated 1978.^3^ The transition was completed with the closure of Forensic Psychiatric Hospitals and replaced by small scale therapeutic facilities (Residenze per la Esecuzione della Misura di Sicurezza) (REMS) established by Laws 09/2012 and 81/2014.^4^ The public mental health system (MHS) in Italy is totally free of charge for users and is funded by the State through the Regional administrations. The MHS is organized uniformly in Italy in a series of Mental Health Departments (MHD). Each MHD comprises one or more networks of Community Mental Health Centers (CMHC), the backbone of the system, supported by related semi-residential facilities (SRF), such as Day Centers (DC) and Community Day Hospitals (CDH), and Residential Facilities (RF) with a maximum of 20 beds; the network includes hospital-based services closely linked to all other services, including General Hospital Wards (GHPW) for acute admissions, with an average of 15 beds, and Day Hospitals (DH). Hospital-based services also include University Psychiatric Units, cofunded by the Italian Government, which often operate in conjunction with MHDs, as well as private Psychiatric Clinics making an agreed proportion of beds available to mental health services. The latest official data for Italy dated 2020^5^ indicated a total of 134 active MHDs with 1299 CMHC, 811 SRF, 1949 RF (26 288 beds, 5.2 × 10.000 inhabitants), and 328 GHPW (4156 beds, 10.5 × 100.000 inhabitants). More than forty years after the Reform, Italian psychiatry continues to rely on a nationwide community care system, although featuring a marked variation in the level and quality of services provided throughout the country.^6^ However, some controversy relating to the system remains,^7^ with particular regard to future trends.^8^ Although support for an ongoing reinstitutionalization is lacking, a series of concerns have been raised with regard to the overall quality of care in some areas of Italy.^6^ The overall balance of the Reform has been generally positive, although a series of challenges, such as the excessive burden on mental health due to a progressive increase in the number of patients followed, diversification in needs of care, and progressive lack of resources have emerged. Although opinions may differ, the Italian psychiatric reform undeniably represents one of the most radical attempts to overcome the practices of custodial psychiatry.^9^ However, more recently, Italy seems to have lost creativity, displaying an increasing neglect of mental health issues, likely due to an ongoing economic and cultural crisis.^10^

## Pandemic Policies Implemented in Italy

On January 31, 2020, the Italian Government declared a Public Health Emergency of International Concern, which has continued to be in place following a series of extension decrees until March 31, 2022. On March 21, 2020, a nationwide lockdown was implemented, which ceased on May 18, 2020. To limit the spread of the COVID-19 infection, once lockdown had ceased, a series of measures were introduced for use based on evolution of the pandemic (ie voluntary or compulsory home confinement, restriction on gatherings of large groups, cancellation of all public events, and a series of domestic and international travel restrictions). As no specific regulations were enforced by the national authorities with regard to the mental health system, each psychiatric department took action as deemed appropriate for the local situation.^11,12^ Several Regional Authorities, including Lombardy, the region most heavily affected at the start of the COVID-19 pandemic, endorsed the availability of guaranteed mental health services, providing occupational and health and safety recommendations for patients and hospital staff, including support for telemedicine activities and remote psychosocial interventions.^13^ Recommendations for mental services were issued by the Italian Society of Psychiatry (SIP)^14^ and the Italian Society for Psychiatric Epidemiology (SIEP).^15^ A second set of recommendations on how to resume activities was issued by the SIP to cover the postacute phase of the pandemic.^16^ A National Vaccination Program set up by the Italian Government in March 2021, identified a series of high priority categories, including subjects affected by “severe disabilities”,^17^ made up solely of people with severe mental disorders who were deemed to have a “severe handicap”. However, thanks to the involvement of individual Mental Health Departments, all patients affected by severe mental disorders were rapidly vaccinated in some Italian regions.

## Impact of the Pandemic on the Mental Health System

Reports describing the state of the psychiatric care system at the onset of the pandemic focused largely on those Italian Regions most heavily affected at the onset of the pandemic. Fusar Poli et al^18^ described the situation in Lombardy and Veneto,^18^ where some psychiatric units had been resized either due to staffing shortages or following reassignment of beds for use by COVID-19 patients, whilst others had dedicated entire inpatient units for occupancy by psychiatric COVID-19 patients; regular outpatient and residential activities were described as being fully operative, although semiresidential clinics, day centers, and day hospitals carried out limited activities or were closed. Patients frequently canceled scheduled in-person appointments, partly due to understandable concerns over infection, resorting to phone appointments and e-communications where possible. De Girolamo et al^19^ reported the main changes in activities performed in the majority of hospitals throughout the 27 Mental Health Departments in Lombardy with entire wards, including GHPWs, being reorganized to admit patients with COVID-19, and staff working in GHPWs frequently being diverted to COVID-19 wards; difficulties in adopting safe practices and isolating hospitalized COVID-19 psychiatric patients were also reported.^19^ The majority of community day facilities were temporarily closed, with many CMHCs limiting intervention to the most urgent cases and drastically reducing home visits.^19^ With the aim of assessing the state of Mental Health Departments in Italy during the first wave of the pandemic, a national survey was conducted by the Italian Psychiatric Association on CMHCs and GHPWs using two specific online questionnaires circulated to the Chiefs of all Italian Mental Health Departments (MHDs).^20,21^ Data were collected from 71 (52.9%) of the 134 CMHCs and 107 (32.6%) of the 318 GHPWs. The results of the survey highlighted how the pandemic had led to a drastic reduction in standards of care, as illustrated in tables 1 and 2. Bearing in mind that activities undertaken by CMHCs, semiresidential and residential facilities could only be verified for 54% of mental health departments, and only one-third of all GHPWs provided data relating to inpatient units, these samples should not be considered fully representative of all Italian services.

**Table 1.** Summary of Data Relating to Community Mental Health Centers, Semiresidential and Residential Facilities^a^ in Italy During the COVID-19 Outbreak (Refs.^20,21^)

	*N*	%
*CMHCs*		
Closed	10	14
Restricted access hours	18	27
Urgent interventions on site maintained	71	100
Scheduled visits (only for urgent cases) remote	63	89
clinical monitoring	64	76
Individual psychotherapies maintained	33	46.4
group psychotherapies maintained	4	5.6
Interventions for compulsory admissions maintained	71	100
Increase of violent acts registered	15	21
*Semiresidential and residential facilities*		
DC closed	60	85
CDH closed	55	88
RF closed	0	0
New admission to RF suspended	55	77.4
Discharge from RF suspended	59	83

*Note*: CMHC, Community Mental Health Centers; DC, Day Centers; CDH, Community Day Hospitals; RF, Residential Facilities.

^a^Values refer to data reported by MHD respondents.

**Table 2.** Summary of Data Relating to General Hospital Psychiatric Wards in Italy During the COVID-19 Outbreak (Refs.^20,21^)

	*N*	%
Closed units	15	13.0
Units with decreased number of beds	34	32.0
Units allowing scheduled admissions	39	36.4
Units with decreased admissions	94	87.8
Units with increased compulsory admissions	9	8.4
Units with decreased psychiatric consultations for other hospital units	74	69.0
Units with increased violent acts among inpatients	9	8.4

Quantitative data on inpatient admissions are reported in three studies focused on GHPWs in various Italian regions^22^ and in two regions in Northern Italy, Lombardy^23^ and Friuli Venezia-Giulia.^24^ The main findings of these studies (table 3) indicate an overall decrease in hospital admissions, particularly in the elderly (>65 yrs), and an increase of log-stay admissions (>14 days), only detected during lockdown, with an increase of suicidal ideation during the postlockdown period.

**Table 3.** Studies of Inpatient Admissions in Italy during the COVID-19 Pandemic

Authors, Ref	Units Included in the Study	Catchment Areas (Localization, Population Served)	Methods	Main Findings
Boldrini et al^22^	12 GHPW	Northern Italy (3.7 million inhabitants)	Comparison of admission across pre-lockdown (PL) periods (March–June 2018; March–June 2019), lockdown (LD) period (March–April 2020) Post-lockdown (PsL) period (May–June 2020)	Decrease of admissions (−41%) during the LD vs PL periods No significant changes in PsL compared to LD Significant decrease in admissions among the elderly (/>65 yrs) during LD (−40%) and PsL (−28%) Increase of long-stay admissions (>14 days) during LD (+63%) and decrease during PsL (−39%) Increase of suicidal ideation (+35%) in the PsL compared to PL periods
Clerici et al^23^	7 GHPW	Lombardy Region (1.4 million inhabitants)	Comparison of admissions over the 40-day period post COVID-19 outbreak (PsO) (21 February–31 March 2020) and the 40-day pre-Outbreak (PrO) period compared to the same time periods in 2019	Decrease in admissions in PsO (−25.7%) vs PrO and (−31.3%) compared to 2019 Significant Decrease (*P* < .001) only for voluntary admissions Significant (*P* < .001) decrease (−55%) only for affective disorders
Castelpietro et al^24^	3 GHPW	Friuli Venezia Giulia Region, 1.2 million inhabitants	Prevalence, incidence, and hospitalization rates during the first four months of 2020, compared to the same period in 2019	Decrease in Voluntary admission rates from 7.53 to 5.98 per 100 000 inhabitants in 2019 and from 6.27 to 3.28 hospitalizations per 100 000 inhabitants in 2020 Compulsory admission rates slightly increased during the first four months of 2019 and 2020, but this rise was not significant

A series of specific studies were conducted to evaluate psychiatric consultations conducted by Emergency Departments (ED) of General Hospitals and Urgent Consultations at CMHCs (table 4). The main findings report a general decrease in ED psychiatric consultations during the pandemic, mainly due to a lower number of requests relating to subjects with depression, adjustment disorders, and suicidality, but featuring a rise in cases of aggression. On the contrary, a rise in number of consultations was registered for patients with a history of previous psychiatric hospitalizations, and a general increase in drug prescriptions (in particular antipsychotics and benzodiazepines) was detected as the outcome of psychiatric consultations. A comparison between the lockdown vs postlockdown period revealed a slight increase in consultations (in particular suicidality) during the postlockdown period. Emergency interventions at CMHCs during the pandemic increased, particularly in enlisted patients; however, the most frequent outcome of urgent intervention was home discharge, indicating a tendency to avoid hospitalizations. However, no significant changes were registered in compulsory admissions as an outcome of urgent psychiatric consultations either by Hospital ERs or CMHCs during the pandemic. In general, in an extensive public health service such as is present in Italy, emergency service provisions seem to have continued to meet the mental health needs of the population during the pandemic.

**Table 4.** Studies of Psychiatric consultations in Emergency Departments in Italy During the COVID-19 Pandemic

Authors, (Ref)	Units Included in the Study	Methods	Main Findings
Stein et al^25^	Emergency Dept-San Paolo Hospital (Milan)	Comparison of psychiatric consultations during lockdown (9 March–3 May 2020) and over the previous 2 months (13 January–8 March with psychiatric consultations performed over the same 16 weeks the previous year (13 January–3 May 2019	ED consultations for mental-health-related conditions reduced during lockdown by almost 50%; No variations in the corresponding period of 2019. decrease throughout all diagnostic categories with the exception of personality disorders, alcohol- and substance-abuse disorders, and trauma- and stressor-related disorders
Balestrieri et al^26^	Emergency departments of nine Italian hospitals (4 located in Lombardy Region)	Comparison of psychiatric consultations during the lockdown and postlockdown periods of 2020 (March 9, 2020 and June 30, 2020) and the equivalent periods of 2019.	37.5% decrease in the number of consultations during the lockdown period and 17.9% after lockdown 34.9% decrease in the number of patients examined during lockdown and 11.2% after lockdown. Higher percentage of patients having previous psychiatric hospitalizations was reported during the lockdown period (61.1 vs 56.3%) and a lower percentage after lockdown (59.7 vs 64.7%). 3.4% decrease in consultations for suicidal ideation and planning during lockdown and subsequent increase for ideation, planning, and suicide attempts after lockdown Increase of antipsychotic (5.2%) and benzodiazepine (4.1%) prescriptions during lockdown Higher number of compulsory hospital admissions after lockdown compared to 2019
Capuzzi et al^27^	Psychiatric emergency rooms of Department of Mental Health ad Addiction, University Hospitals in Desio and MHA of Monza, Lombardy Region	Comparison of psychiatric consultations during phase 1 of lockdown (21/23 May 2020) compared to the same period in 2019	58% decrease in emergency psychiatric consultations during lockdown compared to the corresponding period in 2019 (*n* = 388). Consultations for depressive and adjustment disorders lower in 2020 than in 2019, with consultations for OCD more prevalent during lockdown than 2019 Rate of hospitalizations after emergency consultation significantly higher during lockdown (53.3%) compared to 2019 (42.5%)
Di Lorenzo et al^28^	Emergency Rooms in the 2 General Hospitals in the City of Modena Emilia-Romagna Region	Comparison of urgent psychiatric consultations (UPCs) carried out in emergency rooms (ER) from March 1 to August 31, 2020 with those conducted from March 1 to August 31, 2019	Decrease of UPCs in 2020 (*n* = 476) respect to 2019 (*n* = 602) Reasons for UPC: a lower rate of suicidal behavior (ideation, attempts) was detected during the Pandemic (17.3% in 2019 and 14.1% in 2020) as well as manic episodes (8% in 2019 and 2.9% in 2020); a higher prevalence was found for aggressiveness during the pandemic (3.8% in 2019 and 10.7% in 2020) as well as maladjustment disorders (2% in 2019 and 7.8% in 2020). Outcome of UPC: Lower number of psychiatric hospital admissions (mainly voluntary) in 2020 (21.1%) than 2019 (27%),
Di Lorenzo et al^29^	Community Mental Health Centre, City of Modena, Emilia-Romagna Region	Comparison of urgent psychiatric consultations (UPC) during the coronavirus outbreak from 1 March to 31 August 2020, with the same period in 2019.	Increase in UPC during the pandemic compared to 2019 (*n* = 811 vs 656) Increase in mean daily number of UPC in 2020 (5.3) compared to 2019 (4.07) UPC more frequently required by patients already in charge of local outpatient services in 2020 than in 2019 Outcomes: more frequent discharge at home in 2020 (66% vs 57% in 2019); drug prescriptions, significantly less frequent in 2020 (55% of cases) compared to 2019 (72%)

Finally, there is a paucity of published data relating to the psychiatric care of patients detained in prisons or residents in REMS (Residences for the Execution of Safety Measures); for both these populations, psychiatric care is provided by MHD mental health professionals. di Giacomo et al^30^ published the only Italian report on psychiatric care in a State prison located in the city of Monza in the Lombardy region. The Authors reported a similar overall number of registered consultations in January, February, and March 2019 compared with the same months in 2020 (approx. *n* = 4200), although the diagnosis of patients requiring mental health consultations had varied. Over the first trimester of 2019, the majority of psychiatric consultations were requested by individuals with addiction and personality disorders (25% and 29%, respectively), with similar rates in 2020 (27% and 31%, respectively), whereas during the same period in 2020 the rate of requests by patients with anxiety or depression disorders was significantly reduced (21% vs 14%; *P* = .035), Moreover, no suicides, strictly monitored in prisons due to a higher prevalence of self-harming behaviors among prisoners, were registered in 2020 (vs one case in 2019) (*n* = 4222). Scarpa^31^ published the only narrative report relating to forensic mental health in Italy during the Covid-19 pandemic, describing how admissions and discharges were significantly reduced during lockdown as access to both forensic REMS and nonforensic residential facilities was heavily restricted; according to the author, the latter frequently led to a refusal to admit patients discharged from REMS to a nonforensic residential facility, a common measure adopted by the Courts. In other terms, all systems of forensic and nonforensic residential facilities were essentially “frozen” during lockdown.

## Discussion

The present article highlights the impact of COVID-19 on the mental health care system in Italy, providing epidemiological data for the Italian population derived from studies conducted during the pandemic. However, the response of mental health services described in published articles focuses prevalently patient contacts, with observations varying across regions and different stages of the pandemic, while very little information is available on clinical outcome.

A drastic reduction in standards of care was observed at all levels of MHS, including acute unit wards, day facilities, residential facilities, home visits, and CMHCs, emphasizing the need to remodulate psychiatric services in line with national lockdown regulations to prevent spread of the pandemic. These data suggest how Italian MHS – as the majority of other countries – appeared to be rather unprepared for this emergency, thus indicating an urgent need for adequate supplementary national plans to prevent psychological distress in both people affected by severe mental disorders and healthcare workers (HCWs),^19^ and to preclude similar events from disrupting health systems in the future.^21^

Voluntary hospital admission rates for psychiatric patients were significantly reduced during the pandemic, with hospitals considered to be a hot-bed of potential infection,^22–24,28^ whilst compulsory admission rates were seemingly unaffected.^23,24^ Furthermore, ED psychiatric consultations were significantly decreased^25–28^ for the majority of psychiatric diagnostic categories, with the exception of personality disorders, substance use disorders, and trauma/stress-related disorders^25,26^ and OCD.^27^ However, the decrease in admission rates was counterbalanced by increased rates of urgent psychiatric consultations amongst users of psychiatric residential treatment facilities^27^ or patients attending local outpatient services.^28^ These observations emphasize how the disruption of normal treatment levels in residential and outpatient settings may have impacted on the clinical condition of patients with mental disorders.

These data moreover suggest the need to implement and increase, both at local and national level, MHS organization and standards of care in order to protect and support the mental health of patients with severe mental disorders, of HCWs, and the general population with an aim to preventing a rapid spread of a potential “pandemic of mental disorders”.

Specific issues however have affected the reorganization of MHS during the pandemic. At the onset of the pandemic, and more recently during subsequent “waves”, entire GH wards, including several GHPWs, have been reorganized to admit patients with COVID-19,^19^ leading to a series of concerns over the management of acute COVID+ cases in inpatient units.^21^ When faced with hospitalized COVID-positive psychiatric patients, clinicians should bear in mind that COVID-19 infection may represent a condition of greater vulnerability capable of negatively influencing preexisting medical and psychiatric disorders, producing a pharmacological interaction between psychotropic drugs and antibiotics or antiviral drugs prescribed in the treatment of COVID-19.^32^ Clinicians should take into account evidence-based practical recommendations on the optimal management of drug prescription, based on safety and tolerability profiles of psychotropic medications, in both psychiatric patients with concomitant COVID-19 infection and in COVID-19 patients displaying psychiatric disorders to avoid exacerbating the psychiatric condition and vice versa.^33^ This issue is of particular relevance due to the current lack of structured guidelines and the availability of scarce evidence^34^ of an optimal form of clinical management for these patients.

Several reports have highlighted how people with preexisting mental disorders might be at higher risk of contracting SARS-CoV-2 infection,^35,36^ with an increased risk of worse outcomes than those with no mental health issues.^37^ These findings emphasize the need to provide elevated standards of care which is not confined solely to the behavioral management of psychiatric patients with COVID-19 disease requiring GH admission. Feasibly, specialized psychiatric hubs should be created in GHs for the purpose of managing concomitant conditions of acute psychiatric decompensation and SARS-CoV-2 infection. To date, very few specialized units have provided this type of intervention at both a regional and national level, to manage this new kind of “comorbidity” ^13^. On the other hand, in the absence of this separation, a considerable overload of the MHS could be derived from longer-stay admissions ^22^ of psychiatric patients with concomitant and complicated COVID-19 infection. Furthermore, for these comorbid patients, the possibility of discharge may be complicated by the lack of adequate familiar or social support, thus further lengthening hospitalization times and subtracting resources that could be dedicated to the management of cases of acute decompensation. Furthermore, the suggestion to create specialized hubs for patients in acute crisis with concomitant COVID-19 infection may be advised in order to limit the risk of COVID-19 diffusion during hospitalization. Indeed, patients presenting with manic or psychotic states admitted to acute wards are generally free to move about and interact with other patients, making it difficult to isolate COVID-19 cases, even more so in psychiatric facilities with shared bathrooms and communal spaces. Moreover, as mental health providers are in close contact with patients with frequent therapeutic interactions, an increased risk of infection of healthcare staff should also be considered, potentially resulting in the closure of acute care facilities or a reduction of provided services. To this extent, a strategy to maintain hospital capacity for SMI patients with COVID-19 might lie in the reallocation of resources differentiating the pathways of care with the creation of specialized hubs. In recent months, we have been facing a new wave of the pandemic,^38^ with higher hospitalization rates,^39^ thus implying a risk of further delay of GH accessibility for psychiatric patients with COVID-19 disease. As patients with severe mental disorders are at higher risk of COVID-19 sequelae due to frequent somatic comorbidities (such as hypertension, obesity, diabetes mellitus, respiratory, and cardiovascular diseases) and an unhealthy life-style (smoking, substance and alcohol misuse),^40–42^ MHDs should place pressure on the NHS to ensure that all patients with severe mental disorders are vaccinated, even on a compulsory basis, against COVID-19, rather than merely relying on isolated interventions by some far-sighted directors.

Psychoeducation programs administered by mental health workers should not be limited to the sole management of psychiatric disorders but should include coping strategies to address the psychological consequences of social isolation and quarantine, providing encouragement and creating a supportive environment^43^ to reduce high levels of stress and expressed emotions.^19^ As patients’ psychopathological status might impact negatively on their ability to follow safe hygiene measures (ie, social distancing, hand washing, mask wearing, and other behavioral norms aimed at reducing the risk of infection),^18^ psychoeducational interventions should provide basic instructions and encourage the psychiatric population and their relatives to adopt these measures, in addition to intervening to reduce stigma and discrimination over the treatment and care of psychiatric patients with COVID-19.^44^ Quarantine and social isolation are known contributors to stress and increase of risky behaviors (ie, online gambling, unsupervised use of social media and substance misuse)^37^; psychoeducation therefore should also focus specifically, particularly amongst youths, on preventing psychosocial drifts that may occur when young people find themselves deprived of structured social support and/or are trapped in dysfunctional family contexts.^42,45^

To guarantee continuity of care for at-risk patients during the pandemic and address some of the issues discussed, a valuable response is represented by telepsychiatry (TP) with remote video/phone conferencing or scheduled visits, online blended or coached psychotherapies, and self-help therapies provided through mobile apps.^42,46^ In Italy, these means have provided a way to ensure clinical monitoring, psychological support, teaching of safety measures, amending pharmacological treatments, and collecting information on users and caregivers’ physical health,^20^ thus facilitating the monitoring of patients’ physical and mental health. Generally speaking, TP has met with good patient acceptability, particularly due to the possibility of more frequent and better targeted contacts with mental health staff and reduced waiting times, resulting in a decrease in appointment cancellations, and improving clinical monitoring and continuity.^42^ Moreover, TP is a promising instrument for use in implementing the MH care system in the forensic population, thus overcoming limitations imposed by patients’ prison confinement. Additionally, several reports have emphasized the comparable validity, reliability, and potential effectiveness of TP with interventions conducted in face-to-face services,^46,47^ although concerns have been raised with regards to privacy issues and, particularly, usefulness in emergency situations, including acute psychotic symptoms or self-harming attempts.^46,48^ Although TP and other technological innovations are valuable instruments in ensuring continuity of care for psychiatric populations, several Authors have highlighted that TP should be considered an integrative tool rather than a substitute for routine clinical practice, suggesting that once the pandemic ends, TP interventions should continue to be used and further implemented, integrating them with numerous operational and consolidated clinical practices.^20,42,46^ An important suggestion recommends the provision, at national level, of specific skill training for all mental HCWs in order to render the application of e-mental health tools as feasible as possible.^19^

TP services, including those providing psychoeducation programs, may also be particularly useful in supporting mental-health care workers during and after the pandemic,^46^ for the specific aim of preventing potential breakdowns that would produce an inevitable domino effect on the entire health care system, largely impinging on continuity and quality of care. Indeed, during the early stages of the COVID-19 pandemic, HCWs experienced several forms of psychological distress and adverse mental health outcomes, including higher prevalence of anxiety, depression, insomnia, burnout, acute-stress, and posttraumatic-stress symptoms (PTSSs).^49–54^ Among these reports, Conti et al^54^ conducted one of the largest survey-based studies aimed at exploring the mental health status and psychological care needs of 933 HCWs in Italy during the COVID-19 outbreak, reporting how more than one-third of the sample reported an explicit need for psychological support. The Authors also found that the majority of the sample was affected by somatization, with younger participants and female care workers experiencing higher levels of anxiety and somatization symptoms than men, and nurses more than physicians.^54^ Accordingly, health-care worker teams should be encouraged to support and monitor each other, and team leaders should be trained to identify signs of psychological distress among workers. Moreover, as stigma related to mental health concerns also seem to affect help-seeking amongst HCWs, peer counselling services for clinical staff might also be useful.^37^ Local and national institutions should further invest in mental health support providing both immediate and long-term monitoring and psychological assistance for HCWs, devoting particular attention to women’s mental health, developing specific hotlines or telephone consultation programs to support psychological HCW well-being, reduce stress, and prevent mental exhaustion.

In conclusion, in view of the significant negative impact produced by the COVID-19 pandemic on the lives of people with severe mental disorders and their families, the well-being of health professionals and the organization of the national and local health services, and given the increasing rates of infection registered during current “waves”, more concerted efforts are needed at an institutional level to reduce the medical and socio-economic burdens of the pandemic, responding effectively and in a timely manner to all the issues outlined herein.
